# The GRACE video-telehealth project protocol: a mixed-methods study to improve quality, safety and acceptability of video-telehealth in Australian general practice and residential aged care

**DOI:** 10.1136/bmjopen-2025-110642

**Published:** 2026-04-29

**Authors:** Ann Carrigan, Melissa T Baysari, Georgina Luscombe, Brendan McCormack, Amy Von Huben, Vaibhav Tyagi, Richard Taggart, Jill Nash, Simon Willcock, David Wilkinson, Stephen Barnett, Andrew Bonney, Joel J Rhee, Christopher Pearce, Fiona Robinson, Timothy F Chen, Sanjyot Vagholkar, Heather Russell, Ai-Vee Chua, Ramon Shaban, Soumya Soumya, Agnivo Sengupta, Meredith Makeham

**Affiliations:** 1 Digital Health Human Factors Research Group, Sydney School of Nursing & Midwifery, Faculty of Medicine and Health, The University of Sydney, Sydney, New South Wales, Australia; 2 School of Rural Health, Faculty of Medicine and Health, The University of Sydney, Orange, New South Wales, Australia; 3 Sydney School of Nursing & Midwifery, Faculty of Medicine and Health, The University of Sydney, Sydney, New South Wales, Australia; 4 Leeder Centre for Health Policy, Economics & Data, Faculty of Medicine and Health, The University of Sydney, Sydney, New South Wales, Australia; 5 eHealth NSW, Sydney, New South Wales, Australia; 6 North Sydney Primary Health Network, Sydney, New South Wales, Australia; 7 Primary Care, Faculty of Medicine, Health and Human Sciences, Macquarie University, Sydney, New South Wales, Australia; 8 Centre for Healthcare Resilience and Implementation Science, Australian Institute of Health Innovation, Faculty of Medicine, Health and Human Sciences, Macquarie University, Sydney, New South Wales, Australia; 9 Graduate School of Medicine, University of Wollongong, Wollongong, New South Wales, Australia; 10 Discipline of General Practice, School of Clinical Medicine, University of New South Wales, Sydney, New South Wales, Australia; 11 Sydney Medical School, Faculty of Medicine and Health, The University of Sydney, Sydney, New South Wales, Australia; 12 Sydney Pharmacy School, Faculty of Medicine and Health, The University of Sydney, Sydney, New South Wales, Australia; 13 Western NSW Primary Health Network, Orange, New South Wales, Australia; 14 Sydney Infectious Diseases Institute, Faculty of Medicine and Health, The University of Sydney, Westmead, New South Wales, Australia; 15 Research and Education Network, Western Sydney Local Health District, Westmead, New South Wales, Australia; 16 New South Wales Biocontainment Centre, New South Wales High Consequence Infectious Disease Advisory Service, Westmead, New South Wales, Australia; 17 Community and Primary Health Care, Faculty of Medicine and Health, The University of Sydney, Sydney, New South Wales, Australia

**Keywords:** Health Services for the Aged, General Practice, Nursing Homes, Patient-Centered Care, Telemedicine

## Abstract

**Abstract:**

**Introduction:**

Access to safe, high quality, acceptable and sustainable general practice (GP) and primary care services is essential to improved health outcomes and quality of life for people living in residential aged care homes (RACH). There are, however, critically low levels of service availability and a decline in GPs providing RACH services globally, suggesting there is an urgent need for safe and effective models of care. Telehealth, delivered as part of a holistic model of care, offers a solution to address this gap but comprehensive, person-centred research is needed to directly assess its effect on safety and quality of care in RACH settings.

**Objectives:**

This collaborative 4-year project (General practice and Residential Aged CarE: GRACE video-telehealth) will (1) scope current telehealth models of care and their acceptability and person-centredness, including identifying the barriers and enablers experienced by RACH residents, carers, staff, GPs and practice managers; (2) co-design a best-practice model of care with an accompanying suite of digital resources and education materials to improve the uptake of video-telehealth; and (3) implement and evaluate this best-practice model of care.

**Methods and analysis:**

This is a mixed-methods study of residents, carers, RACH staff, GPs and their practice teams that will be conducted across New South Wales, Australia. This protocol describes a staged approach across three phases. In Phase 1, we will collect baseline measures of the frequency of telehealth use in GP practices and RACHs, clinical outcomes (eg, hospitalisations), questionnaires to measure person-centred care, satisfaction and usability of telehealth and qualitative observations and semi-structured interviews. In Phase 2, we will conduct workshops to co-design an intervention that will include developing a model of care to support person-centred video-telehealth, with an accompanying online hub of resources and educational materials to facilitate and support its utilisation. In Phase 3, we will implement and evaluate the intervention. Data will be analysed statistically and thematically and synthesised.

**Ethics and dissemination:**

Ethics approval has been obtained from the University of Sydney Human Research Ethics Committee (2025/000340) (human.ethics@sydney.edu.au). Prior informed written consent will be obtained from all research participants. Findings from each phase of the study will be submitted for peer-reviewed publication. Project outputs will be disseminated for implementation more widely across New South Wales and Australia.

**STRENGTHS AND LIMITATIONS OF THIS STUDY**

- A 4-year, staged, comprehensive study of video-telehealth models of care in an aged care context
- Engagement with key stakeholders in government and peak bodies, clinicians including general practitioners, nurses, residential aged care home providers and consumers to scope, develop and evaluate an intervention that is fit for purpose
- The mixed-methods approach using established Human Factors and Person-Centred frameworks will enable a rich synthesis of data from multiple sources
- May not capture the diversity of the characteristics and participant experiences of those within and outside of New South Wales, Australia

## Introduction

 The global population is rapidly ageing, where in Australia the proportion of adults over 65 years has grown by 32% in the past decade,^1^ and the number of older adults aged ≥85 years is projected to double by 2042.^2^ Consequently, there is an unprecedented demand for high quality and safe aged care services,^2^ with admissions to residential aged care homes (RACHs) increasing by 57% since 2013.^1^ By contrast, in the next 10 years, there is an expected reduction in access to general practitioner (GP) services, with demand predicted to increase by 38%.^3^ Additionally, the substantial out-of-pocket costs GPs incur when visiting RACH residents in person, often without adequate reimbursement from Medicare, Australia’s tax-funded universal health insurance system, may be contributing to increasing reluctance to provide in-person care. At present in Australia, approximately 5400 GPs^4^ serve over 240 000 residents in 2641 RACHs.^1^ This situation is unsustainable, as demand is predicted to outpace supply, and therefore requires innovative and scalable solutions for delivering safe, high-quality GP services to RACH residents, particularly in regional and remote areas where there is a greater proportion of people aged 65 years and over.^3^ Improved access to GPs for RACH residents leads to improved health outcomes for older people, with Australian Medical Association estimated savings of $A21.2 billion over 4 years (2021–2025) from avoidable hospital admissions, emergency presentations and hospital stays if governments invested sufficiently in health and aged care.^5^ The ageing population presents complex challenges to an already burdened healthcare system, driven by demand for general and specialist services, an increase in the prevalence of chronic diseases and provider shortages.^6^

The Royal Commission into Aged Care Quality and Safety, a major public inquiry established by the Australian Government in 2018, highlighted significant gaps in access to services for aged care residents, with 17–31% of RACH transfers to emergency departments deemed unnecessary.^7 8^ One of its key recommendations was that RACHs should be equipped with the necessary tools and staff to support virtual models of care that include video-telehealth services between GPs and RACH residents.^9 10^ Additionally, Australia’s national reform plan for future-focused primary health care^11^ emphasises virtual care models and person-centred continuity of care,^12^ which could improve access to GP-led services for RACH residents.

Video-telehealth has been recognised as a mainstream reform in GP healthcare, implemented in Australia initially for GP consultations during the COVID-19 pandemic.^13^ By December 2021, video-telehealth became a permanent feature, with 20–30% of all GP visits now virtual,^14^ largely supported by improvements in technological infrastructure and connectivity.^15^ Across all ages, virtual care has been associated with improved medication adherence,^16^ patient knowledge,^17^ satisfaction^18^ and reduced hospital readmissions,^19^ compared with in person care. Recent mixed-methods research found that most consumers and providers viewed virtual care positively, citing improved accessibilty and patient well-being.^15^ However, many participants felt that this model of care was not a “one-size-fits-all” approach, particularly for indivudals with high acuity of care and or limited digital literacy. To overcome these barriers, educational resources such as training on care delivery modes to support the learning of new skills were recommended.^15^ Video-telehealth offers cost effective and timely access to care, which is especially relevant given the gaps in GP-led RACH services^3^ and the ageing population.^2^

In aged care settings, video-telehealth could provide accessible and efficient care, thereby reducing physical and logistical burdens for frail older adults and their carers, and travel time for GPs. This therefore results in a faster clinical response leading to care plan adjustments which can reduce hospital transfers and improve care coordination among supporting staff.^20^ Video-telehealth can also help mitigate risks associated with hospital transfers, such as delirium, falls, medication errors and deconditioning and death.^21 22^ International studies have demonstrated that the addition of routine video-telehealth consultations to usual care can reduce avoidable hospitalisations.^23 24^ However, there is no comprehensive Australian research that directly assesses the impact of GP-led video-telehealth on safety, quality of care and cost-savings in RACH settings. Additionally, GPs recognise the potential benefits of video-telehealth; however, barriers related to implementation (eg, insufficient training), technology (eg, inadequate internet connectivity) and workflow (eg, unpredictable delays when calls are not coordinated well) remain.

Healthcare systems are complex and multifaceted. Understanding the barriers related to implementation, technology and workflow in healthcare, therefore, requires a whole system and human-centred approach. Human Factors (HF), the study of how people interact within systems, provides a way to understand the barriers to video-telehealth by considering the work system elements involved (technology, tasks, people, environment), processes and outcomes,^25^ for example, barriers related to usability of technology.^26^ Additionally, person-centred practice (PCP) ensures that care is respectful, individualised and responds to a person’s needs.^27^ Together, adopting a HF and PCP approach creates digital health environments that are safe, efficient and meaningful which is crucial in aged care settings.

The recent national investments in GP-RACH digital health, along with the capability many GPs now have in conducting government-funded video-telehealth following COVID-19, provides a unique research opportunity. This programme of research represents a timely and essential effort to explore and optimise video-telehealth services for RACH residents. This study will identify current telehealth models (with or without video), optimise them and provide the necessary resources and guidance for their effective implementation in RACHs, possibly supporting safer and higher-quality care for residents. By improving access to GP-led care through video-telehealth, this study will help address the urgent need for new, sustainable models of care that ensure older adults in Australia and globally receive the safe, high-quality care they deserve. Through collaboration with clinicians, RACH staff and Primary Health Networks (PHN), the overall project aims to set a national standard for video-telehealth adoption that benefits residents, healthcare providers and the broader health system.

### Study aims

The research has three key objectives delivered across three Phases.

Aim 1: Phase 1 (pre): To examine and scope the current use of video-telehealth and determine its usability and safety with respect to supporting work process alignment (barriers and enablers), PCP and satisfaction for residents/carers, RACHs, GPs, nurse practitioners (NPs), managers and staff.

Aim 2: Phase 2: To co-design and develop a best practice model of care enhanced by video-telehealth that includes a digital hub of resources and support materials, using co-design methods and implementation strategies that drive adoption of person-centred video-telehealth in GP/RACH settings.

Aim 3: Phase 3 (post): Guided by the RE-AIM framework (Reach, Effectiveness, Adoption, Implementation and Maintenance),^28^ we will implement the intervention developed in phase 2 and measure any change in the uptake of video-telehealth instances, acceptability, usability and safety with respect to supporting work process alignment (barriers and enablers), PCP and satisfaction for residents/carers, GPs, NPs, managers and staff.

## Methods and analysis

### Study design

This is a mixed-methods study of residents, carers, RACH staff, GPs and their practice teams including NPs, nurses and practice management staff. We will adopt a co-design approach involving consumer representatives from across New South Wales (NSW) including rural regions, to ensure that the research is meaningful and relevant.^29^ The study comprises the frequency of telehealth use in GP practices and RACHs, clinical outcomes, surveys including demographic information and questionnaires, and qualitative observations, semi-structured interviews and workshops. In Phase 1 we will examine and scope the use of video-telehealth and subsequently develop and co-design a video-telehealth model of care and hub of resources (the intervention) in Phase 2. Phase 3 will implement and measure any changes in the uptake of video-telehealth instances, clinical outcomes, usability, sustainability, cost effectiveness and safety with respect to supporting work process alignment (barriers and enablers), person-centred culture and satisfaction for residents, RACHs, GPs, NPs and staff.

### Theoretical approach

**Figure 1:**
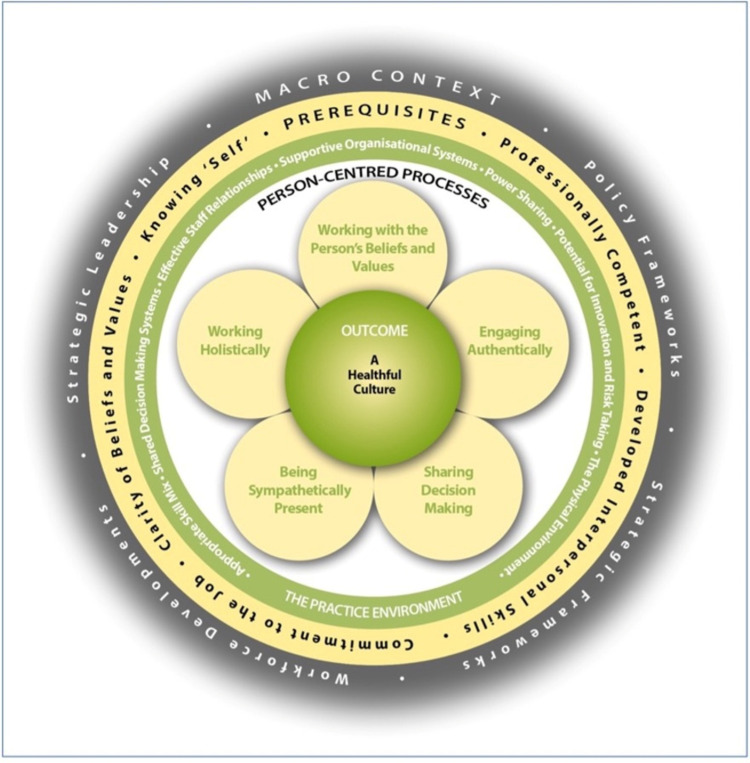
The Person-Centred Practice Framework.

The Person-Centred Practice Framework (PCPF) will be used to measure person-centred culture and acceptability of video-telehealth^27^ (figure 1). Two PCP Inventories^27^ developed using the PCPF and applied in numerous international aged care settings to assess person-centred culture^30^ will be used.

**Figure 2:**
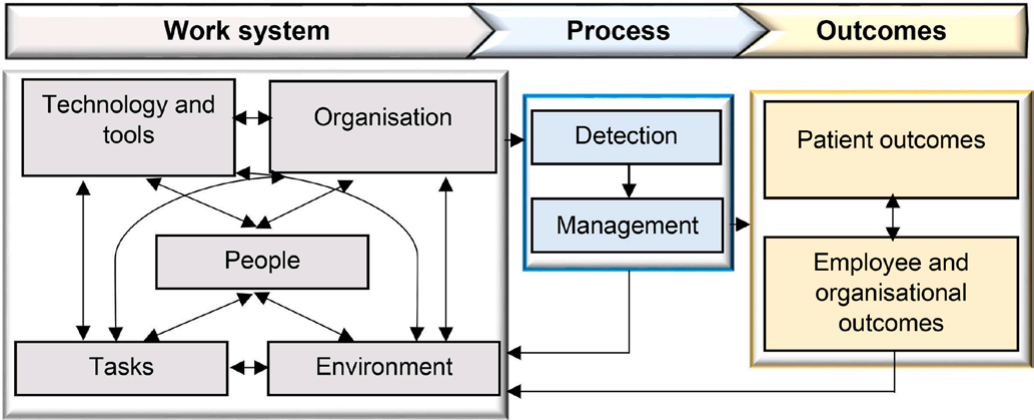
Systems Engineering Initiative for Patient Safety (SEIPS) model of patient journey and safety.

In addition to examining PCP, we will adopt a HF approach to assess the factors that affect patient safety within a complex environment such as aged care. The Systems Engineering Initiative for Patient Safety (SEIPS) model, a well-established HF framework that has been used in patient safety research, will be used to gain a comprehensive understanding of work systems, processes and outcomes when video-telehealth technologies are used^25^ (figure 2).

In Phase 2, the workshop activities will be guided by the Promoting Action on Research Implementation in Health Services (PARiHS) framework,^31^ a commonly used implementation science conceptual model that has successfully been used to implement change in medical and nursing settings and tested with the PCPF, providing a good fit for our GP-RACH settings.^27^

In Phase 3, we will be guided by the RE-AIM framework, an established model that has been widely used in health services research to assess real-world impact of the intervention.^28^ The framework proposes assessing Reach (eg, number of residents involved in the intervention), Effectiveness (eg, resident experience), Adoption (eg, number of GPs using video-telehealth), Implementation (eg, training required) and Maintenance (eg, practices that embed video-telehealth into routine workflows).

### Study setting

**Figure 3:**
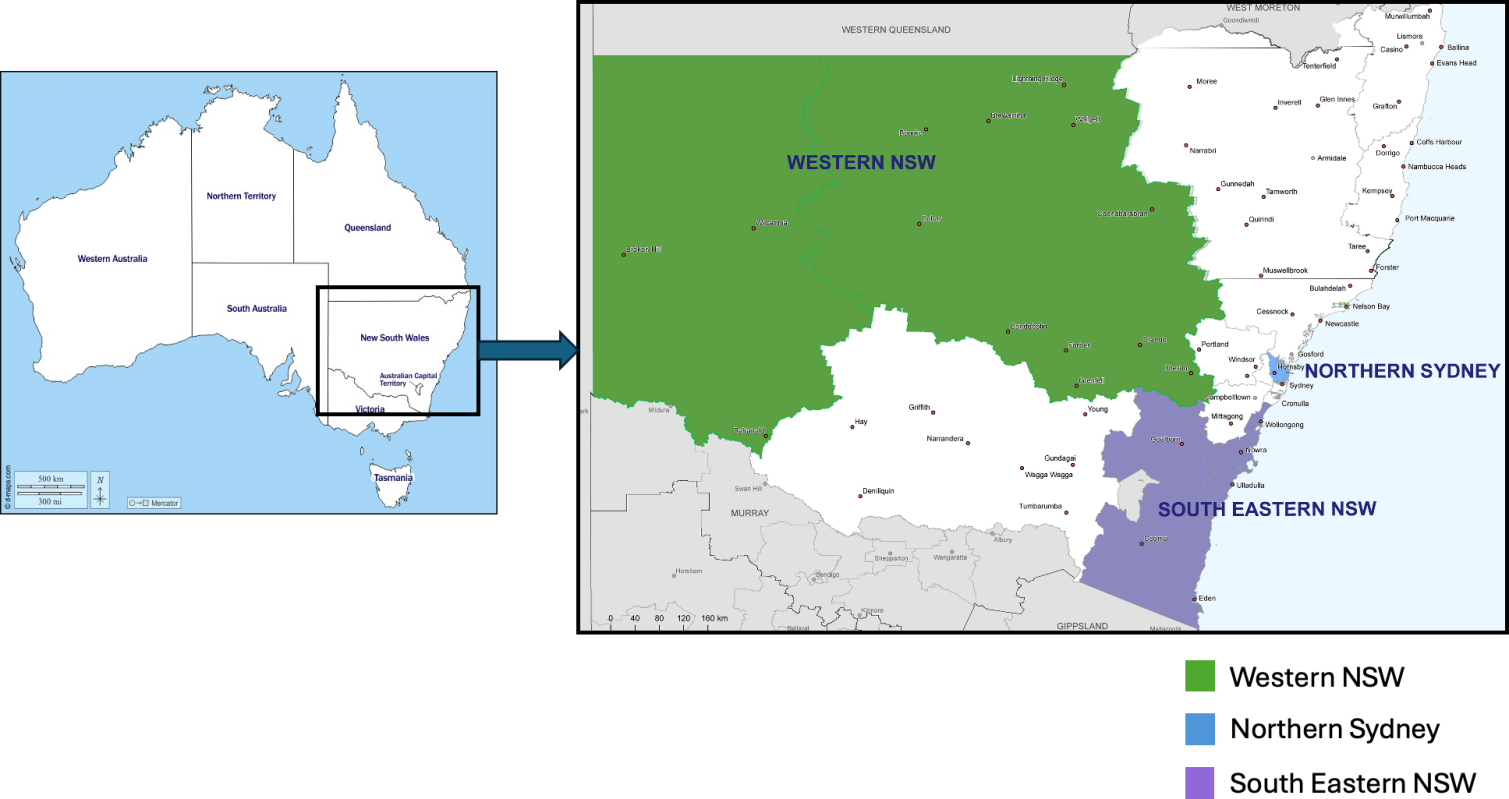
Geographical location of the participating Primary Healthcare Networks within NSW, Australia. NSW, New South Wales.

The research will be conducted from October 2025 to December 2028, in partner RACHs and PHN regions in NSW, Australia’s largest state, selected for regional diversity (figure 3).

These areas are geographically (urban to very remote) and demographically (cultural, linguistic and country of birth) diverse. This diversity shapes healthcare needs and service delivery, requiring tailored approaches specifically for aged care. Here we have defined location based on the Modified Monash Model (MMM) which classifies remoteness and population size on a scale ranging from MM1 (major city), MM2 (regional centre), MM3 (larger rural town), MM4 (medium rural town), MM5 (small rural town), MM6 (remote communities) to MM7 (very remote communities)^32^ (table 1).

**Table 1:** Characteristics of the participating NSW primary healthcare networks

PHN	Size (km^2^)	MM range	General practices (frequency)	General practitioners (frequency)	RACHs (frequency)	Resident beds (frequency)
Northern Sydney	899.9	1, 2, 5	292	1179	100	9000
South Eastern NSW	50 177	1–5	198	780	80	3935
Western NSW	433 379	3–7	109	305	52	3162

MM, Modified Monash; NSW, New South Wales ; PHN, Primary Health Network; RACHs, residential aged care homes.

### Study participants and recruitment

We will aim to ensure representation across key stakeholder groups involved in video-telehealth delivery and receipt of care. Across all three phases, participants will include GPs and NPs, practice managers, RACH staff, including registered nurses, managers and carers. While exact numbers per group will be determined by availability and relevance, we anticipate approximately half of participants will be healthcare providers (GPs/NPs and practice or RACH staff) and half residents and carers. In line with previous research, purposive sampling will be used to achieve diversity in professional roles, care settings and geographic regions, rather than statistical representativeness. We will recruit up to 72 participants across all potential respondents (GPs, NPs, managers, RACH staff, residents) and PHNs accounting for study attrition. We have based this sample size on requiring~24 participants for each phase. This strategy is based on our experience of reaching thematic saturation in similar studies.^33^ We will approach approximately 30 GP practices to seek consent to obtain their de-identified telehealth encounter data with RACH residents.

#### Residential aged care homes

We will include RACH managers, nurses and staff in RACH organisations who deliver or support care for RACH residents with or without video-telehealth. Eligible residents will be required to be able to provide informed consent, be aged 65 years or more and have had experience with a telehealth consultation (phone or video). Residents with cognitive impairment (including mild cognitive impairment or a documented diagnosis of dementia) may be invited to participate where their involvement is deemed appropriate by their usual RACH staff, based on day-to-day knowledge of the resident’s communication abilities and capacity to engage with the study activities. Capacity to consent will be assessed in accordance with ethical guidelines and the resident’s ability to understand, retain and communicate a decision regarding participation. Where a resident is assessed as lacking capacity to provide informed consent, participation will only occur with the consent and support of a legally authorised representative (eg, a carer, family member or person exercising lawful authority).

To support inclusion of residents from culturally and linguistically diverse backgrounds, non-English-speaking residents may participate with the assistance of a carer or family member acting as an informal interpreter, where appropriate and acceptable to the resident. Interpreters will support communication during interviews or questionnaires but will not influence responses. The voluntary nature of participation will be emphasised, and residents and carers may withdraw at any time without consequence.

#### General practitioners

Eligible GPs must hold current Australian Health Practitioner Regulation Agency registration as a Specialist General Practitioner and have experience providing care in RACHs. NPs endorsed with the Nursing and Midwifery Board of Australia, practice nurses and practice managers at general practices offering video-telehealth are also eligible to participate.

All participants will be informed that participation is voluntary, and they will be offered an honorarium (gift card) for each phase of the research in which they participate. GPs will also be offered continuing professional development (CPD) points.

## Data collection

### Phase 1: Examine and scope

#### Quantitative

Telehealth encounters between GPs, NPs and RACH residents will be extracted at tw0 time points: baseline (pre) and post (~6–12 months post implementation) and measured by these methods:

(1) Practice-level clinical software extractions

With GP and practice consent and oversight, de-identified counts of RACH in-person and telehealth consultations will be extracted by the research team in collaboration with practice managers using a standardised query template compatible with major clinical software systems. Practices will be asked to confirm the accuracy of their appointment coding (eg, telehealth vs in-person) and identify any known system limitations prior to extraction.

(2) Administrative datasets (Services Australia and Lumos NSW)

With GP and NP consent, de-identified consultation data (video, telephone, in-person) will be requested from Services Australia (an Australian Government executive agency), and Lumos NSW (an NSW health programme that securely links de-identified GP data with other health system data to generate insights into patient journeys and improve healthcare services), in accordance with their Consented Data Release Guidelines. These datasets include internal validation processes and documentation of known delays or suppression rules. To minimise incomplete records, data will be requested only after the relevant claim periods have closed.

Clinical quality and safety indicators (eg, hospital transfers, falls) from RACHs will also be recorded. No identifiable resident data will be shared.

All participants will complete a demographic survey (eg, age, gender) with GPs/NPs and RACH staff asked additional items such as their professional experience. Two PCP Inventories^27^ developed using the PCPF and applied in numerous international aged care settings to assess person-centred culture^30^ will be administered for GPs, RACH staff and residents/carers in Phase 1 (see online supplemental file 1).

1. Person-Centred Practice Inventory Staff Short-Form (PCPI-S (SF))^34^ measures healthcare providers’ perceptions of their PCP.
2. Person-Centred Practice Inventory Consumer (PCPI -C) measures residents/carers’ perceptions of the person-centred culture.

To measure whether the video-telehealth technology and services delivered are effective and efficient, users (eg, GPs/NPs, residents and carers) will complete the validated Telehealth Usability Questionnaire (TUQ).^35^ The domains related to patient satisfaction with their healthcare treatment will be measured with the validated Short Assessment of Patient Satisfaction (SAPS) scale^36^ (see online supplemental file 1 for the TUQs (GP/NP and patient versions) and SAPS items). To minimise participant burden, questionnaires will be administered in flexible formats (secure online platform or paper), and support to complete questionnaires will be offered to residents as needed. Questionnaires selected for this study have been previously validated in older adult populations^34^ and data collection will be paced to align with participant preferences.

#### Qualitative: observations and interviews

GPs/NPs, practice managers, RACH staff, residents and carers will be observed during video-telehealth consultations to assess usability, work processes (barriers and enablers), person-centred care and satisfaction. Observations will be conducted in-person, with fieldnotes documenting workflows, not individual medical details. Semi-structured interviews will explore telehealth experiences, including what is working, what is not working and workarounds. These will be conducted in person or online, recorded and transcribed (see online supplemental file 2 for RACH staff interview guide as an example).

### Phase 2: Design and development of a digital hub of resources (intervention)

In Phase 2, the outcomes of Phase 1 will inform the co-design and development of a proposed model of care to support person-centred video-telehealth, and an intervention to facilitate and support video-telehealth utilisation will be developed.

Initial review of proposed models and materials will be conducted using a Framework Analysis method (see data analysis) to ensure alignment with the PCPF^27^ and associated principles. Consumer representatives from the three PHNs located in metropolitan, regional and rural settings will be involved across the consultation process including interviews or focus groups via project partner HealthConsumers NSW. They will be invited to review potential support materials for residents and their carers and seek further guidance on the development of the intervention.

### Phase 3: Implementation and evaluation

The intervention developed in Phase 2 will be implemented and evaluated in Phase 3.

**Figure 4:**
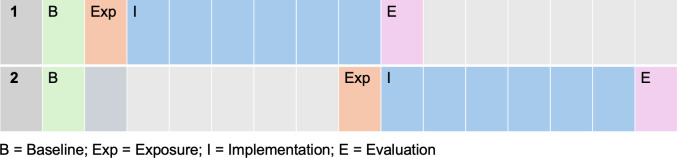
Implementation plan.

The study groups (GPs/RACHs, etc.) will be split into two with Group 1 receiving the intervention first, and Group 2 receiving it after waiting 6 months. The intervention will be implemented supported with guidance over 1 month (exposure). Participants will be offered educational sessions and access to a telephone contact for support and troubleshooting. Evaluation of the intervention using the same outcome measures used in Phase 1 will be conducted 6 months after implementation (figure 4) in addition to the RE-AIM framework. Using this framework we will examine who participates and the representativeness of residents, carers, staff and GP practices (Reach); assess clinical outcomes, resident, carer, staff and GP experiences with the intervention (Effectiveness); consider which GP practices, RACHs and staff choose to take up the model of care (Adoption); evaluate fidelity, adaptations, resource requirements and barriers to delivery (Implementation) and explore whether video-telehealth becomes embedded in routine workflows at both individual and organisational levels (Maintenance).

#### Workshops

We will develop and co-design the intervention involving consumers from each PHN region, partners and end users. The intervention will be a digital hub of resources to support video-telehealth for providers in general practices and staff/residents in RACHs. We will develop implementation strategies within each PHN such as GP-RACH adoption toolkits, education sessions, CPD accredited resources for GPs and nurses, online video resources and other information for RACH residents and carers. Our digital hubs, designed for each PHN region to meet their unique needs, will undergo usability testing and be reviewed by our partners. A consensus will be reached by the full team prior to Phase 3 evaluation.

**Figure 5:**
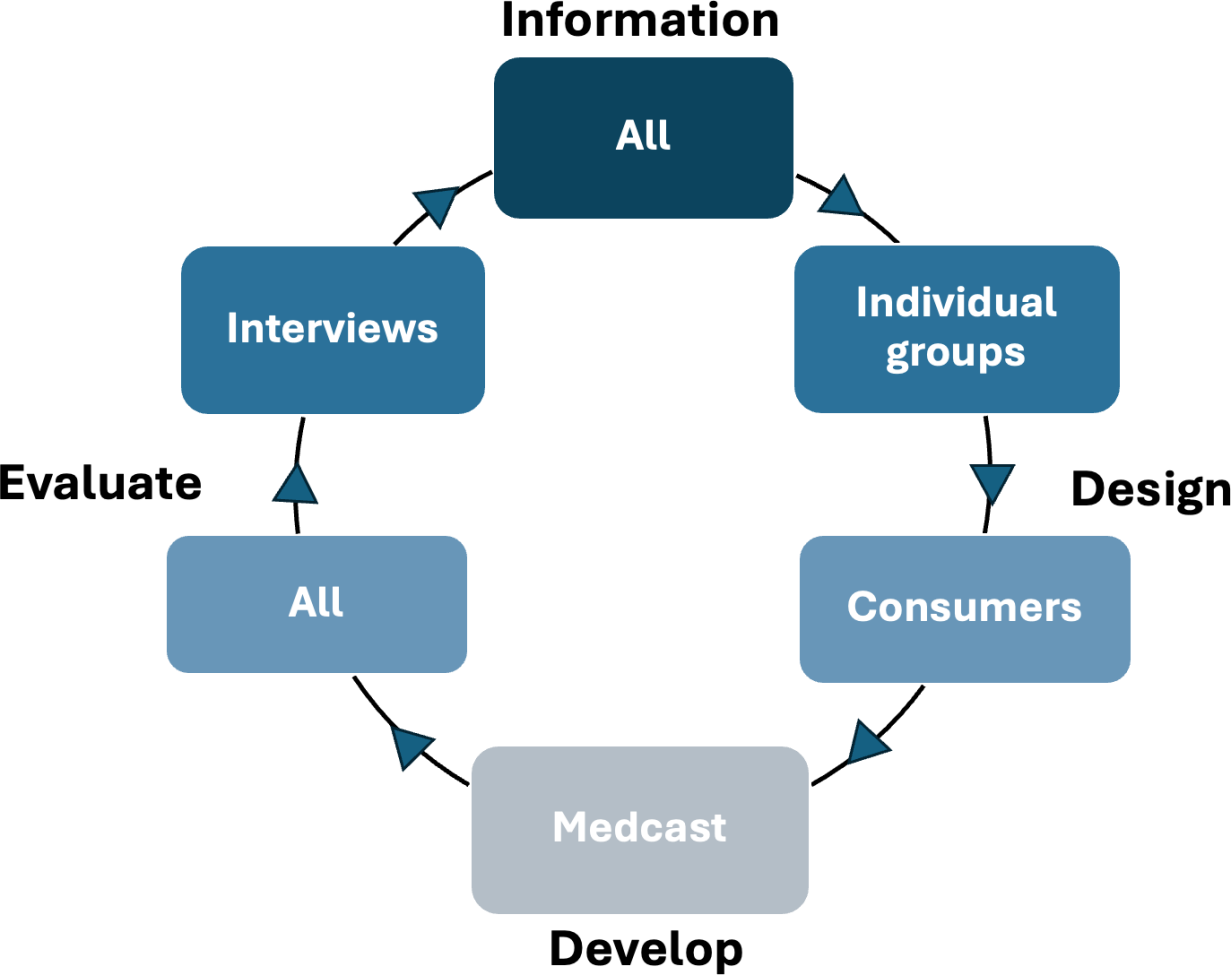
Phase 2 workshops data collection cycle.

For the development of the intervention, we will conduct 3–5 workshops together with providers from GP and RACH settings, residents and their families/carers, and consumers hosted by our partner Medcast, a leading Australian provider of innovative health professional education (https://medcast.com.au/). Medcast will only be involved in the development of the intervention, not its evaluation or data analysis. Workshops will commence with an information session for all groups where we will present the findings from Phase 1. Individual groups and a consumer workshop will follow, with all groups coming together again after the first iteration of the intervention has been designed (figure 5).

The aims of the workshops will be to:

- Present to workshop attendees a summary of the findings from Phase 1 (ie, barriers and enablers and experiences for delivery of video-telehealth).
- Understand participants’ expectations and preferences for delivery of education resources and video-telehealth in the primary healthcare sector.
- Understand expectations and preferences of primary care in delivering video-telehealth to a RACH.
- Co-design education resources that are acceptable to GPs and managers, RACH staff and residents to improve capacity for video-telehealth in primary care.

#### Semi-structured interviews

We will conduct interviews to explore perspectives, experiences and opinions, and usability testing with~3–4 subject matter experts, to evaluate the prototype of education and support resources and materials (the intervention). Interviews will be conducted face-to-face, online or via telephone, and will be audio recorded and will last around 30 min. This process may be repeated if a second prototype is developed based on the feedback obtained in the interviews.

The main outcome of Phase 2 will be a hub of online resources developed by and hosted on Medcast and education materials. The details of the workshop guide are illustrated in figure 5, and outlined in online supplemental file 3.

### Planned data analysis

#### Quantitative (Phases 1 and 3)

De-identified GP consultation data (in-person and telehealth), clinical outcome data, telehealth usability and patient satisfaction data will be aggregated and analysed using statistical software (SPSS).^37^ Descriptive statistics such as frequencies, percentages and means will summarise demographic data and general patterns in telehealth use. Person-centred questionnaire scores will be calculated as averages across staff and consumer ratings for each PCP framework construct score.^27^ For this project, we will map the PCP scores to the PCPF^38^ (see figure 1). We will assess alignment of telehealth models with PCP, in consultation with RACH-experienced clinicians (~n=10–12), followed by full team review.

### Data quality and missing data management

To ensure completeness and reliability across all sources, datasets will undergo structured quality checks, including assessment of missingness, internal consistency (eg, comparing practice-reported counts with Services Australia totals where feasible), and range checks for implausible values. Where discrepancies or missing data are identified, clarification will be sought from the data provider when possible. Irreconcilable inconsistencies will be documented, and analyses will use complete-case data with sensitivity analyses conducted to assess the impact of missingness. All data quality limitations will be transparently reported and considered when interpreting findings.

#### Qualitative

In Phases 1 and 3, two researchers will conduct thematic analysis of observation and interview data in two stages: inductive coding to identify themes, followed by a deductive mapping using NVivo^39^ to the SEIPS framework (people, tasks, tools, environment and organisation).^40^ To enhance rigour, two researchers will independently code an initial subset (~20%) of transcripts using an iteratively developed codebook. After open coding, the team will meet to compare generated codes, resolve discrepancies by consensus and refine codes. The remaining transcripts will be double-coded where codes are emergent or complex; otherwise, a single-coder approach will be used with scheduled peer debriefs and spot-checks to ensure consistent application. Reflexive memos will accompany each analytic cycle to make researcher assumptions explicit and to reduce bias in development of themes.

This will highlight factors influencing telehealth experiences, workflow barriers, technology issues, workarounds and the broader work system (eg, user capabilities, tasks supported, required infrastructure and other practice and RACH work processes). In Phase 2, workshop and interview data will be thematically analysed using open coding to identify themes that inform implementation strategies and the design of a best-practice video-telehealth digital hub of resources (the intervention).

A detailed plan for the analysis will be submitted as a separate protocol which includes economic outcomes where we will assess the costs and outcomes associated with the video-telehealth intervention.

### Multi-methods analysis

The quantitative and qualitative outcomes of Phases 1 and 3 will be used to undertake analyses to determine the effect of video-telehealth on safety and quality of care and its acceptability to residents, carers and visiting clinicians.

### Synthesis and integration of results

The quantitative and qualitative findings will be triangulated, whereby participant characteristics, questionnaire/survey results, observations and interviews will determine what is and is not working when telehealth is used and whether it involves person-centred care among RACH staff, residents, carers and GPs/NPs. It is anticipated that multiple video-telehealth models and work system components may be identified. Thematic analysis and consensus discussions with our multidisciplinary team and partner organisations will determine ‘successful’ video-telehealth models aligned with GP-RACH workflow. The findings will be used to inform the subsequent development of the intervention to improve implementation, adoption and outcomes of the use of video-telehealth in a RACH setting.

## Discussion

Providing timely and quality healthcare for RACH residents in Australia is reaching a crisis point because there is a decline in GPs providing services.^3^ Video-telehealth provides a potential solution and some training options to support this approach are available.^41^ However, the identification of the challenges relating to technology in this context, and safety and quality evidence are needed. Additionally, barriers related to significant financial costs in visiting RACH which are not met by Medicare may mean that GPs are becoming more reluctant to provide ‘in person visits’. Involving consumer advocates and co-design methods, the project aims to scope, design, implement and evaluate resources and strategies for video-telehealth adoption that will benefit RACH residents and carers, staff and GPs, and the broader health system. We will scope the current use of video-telehealth and provide a comprehensive analysis on provider and consumer experiences engaging with video-telehealth. Phase 2 involves the co-design of a digital hub of resources, fit for purpose, to improve the uptake and user experiences using video-telehealth that will be implemented and evaluated in a subsequent phase. Given that the demand for aged care services is predicted to rise significantly^1^ as the older population increases,^6^ this research will provide evidence on alternative models of care that support safe and quality healthcare for this vulnerable population.

The limitations of this study may include the following: There is a risk of selection bias as the study may not generalise to all RACHs and GP practices, only those within the three selected PHNs within NSW. However, we have deliberately ensured that we will capture data from diverse regions including remote regions. The interview questions and workshops will be conducted in English and may not capture the diversity of experiences and views of those in other cultures, settings and locations, which may limit generalisability.

## Expected outcomes

The initial project phases will generate evidence on current use, barriers, enablers and best practice models for video-telehealth in GP and RACH settings. We will develop a digital hub of resources to support implementation, and using evidence-based methods, co-design, implement and evaluate the intervention that promotes person-centred care and work-aligned care. Unlike existing Australian telehealth initiatives in aged care, which have primarily focused on service expansion or feasibility, this study integrates HF methodologies, analysis, measurement of outcomes related to PCP to systematically examine safety, workflow alignment and acceptability of GP-led video-telehealth across diverse RACH settings. Overall, our co-designed model may help reduce inequities and primary care shortages, lowering risks associated with unnecessary hospital transfers. The project will inform future health policy and support evidence-based decisions by GPs and their practices, RACHs, policymakers and funders. Ultimately, it will enhance resident well-being by enabling safe, holistic care at home through video-telehealth.

## Ethics and dissemination

### Consent

The project was peer-reviewed and approved by the University of Sydney Human Research Ethics Committee and all stakeholders. Participation poses no known health or other risks. Findings will be published in peer-reviewed journals, presented at national and international conferences and shared with stakeholders. Participants who expressed interest via the consent form will receive a summary of key results at the project’s conclusion.

### Managing risk/distress

Participation is voluntary, with no known health or other risks. Low enrolment will be addressed through broad, multichannel recruitment and re-advertising if needed. Residents and carers may experience mild distress due to past negative experiences with technology. To mitigate this, the interview purpose will be clearly explained and participants reminded they can withdraw at any time. Interviews will be confidential, with personal data de-identified. Participants may revise responses or request deletion of recording before transcription.

All participants will be informed that their decision to participate or withdraw will not affect their relationship with PHNs, RACHs or the University. To avoid perceived coercion, researchers will only contact RACH staff or GPs after they express interest. Any serious adverse events will be reported to the Ethics Committee and its recommendations followed.

### Confidentiality

Participant information, consent forms and demographics data will be provided in electronic or paper-format. Paper materials will be scanned and then securely destroyed. All participants will be de-identified and assigned a unique ID. Personal contact details will be used only for scheduling and deleted after participation. Electronic data will be stored securely on the University of Sydney Research Data Store, with backups on the University’s OneDrive. Access is restricted to the study team. Data will be retained for at least 7 years and then deleted. No participants will be identifiable in any published outputs.

## Supplementary material

10.1136/bmjopen-2025-110642 online supplemental file 1

10.1136/bmjopen-2025-110642 online supplemental file 2

10.1136/bmjopen-2025-110642 online supplemental file 3
