# Supplementary material for: The GRACE video-telehealth project protocol: a mixed-methods study to improve quality, safety and acceptability of video-telehealth in Australian general practice and residential aged care
Source: BMJ Open. 2026 Apr 29;16(4):e110642. doi: 10.1136/bmjopen-2025-110642 (PMC13141005; doi:10.1136/bmjopen-2025-110642)
Supplement: online supplemental file 1 [file bmjopen-16-4-s001.pdf]

# PCPI-S (SF)

## Instructions for filling the form

This questionnaire consists of statements about different aspects of your professional practice and your work environment. There are no “right” or “wrong” answers. What matters is your honest reflection on how often these practices occur in your daily work.

Please read each statement carefully and select one option that best describes **how frequently the behaviour or experience occurs in your practice**. For each statement, tick *one* of the following response options: Never, Rarely, Sometimes, Often, Always.

**Please respond to all questions.** If you are unsure about a particular item, choose the answer that feels most accurate based on your experience.

## About yourself

Gender (please tick one): ○ Male ○ Female ○ Non-binary ○ Transgender ____________ Other (please specify)

Practice experience (in years): ____________________

Age (in years): ____________________

## Questionnaire (please tick one response for each question)

| | Never | Rarely | Sometimes | Often | Always |
|---|---|---|---|---|---|
| 1. I have the necessary skills to negotiate care options. | ○ | ○ | ○ | ○ | ○ |
| 2. When I provide care I pay attention to more than the immediate physical task. | ○ | ○ | ○ | ○ | ○ |
| 3. In my communication I demonstrate respect for others. | ○ | ○ | ○ | ○ | ○ |
| 4. I pay attention to how my non-verbal cues impact on my engagement with others. | ○ | ○ | ○ | ○ | ○ |
| 5. I go out of my way to spend time with people receiving care. | ○ | ○ | ○ | ○ | ○ |
| 6. I strive to deliver high quality care that is informed by evidence. | ○ | ○ | ○ | ○ | ○ |

Questionnaire (please tick one response for each question)

| | Never | Rarely | Sometimes | Often | Always |
|---|---|---|---|---|---|
| 7. I take time to explore why I react as I do in certain situations. | ○ | ○ | ○ | ○ | ○ |
| 8. I challenge colleagues when their practice is inconsistent with our team's shared values and beliefs. | ○ | ○ | ○ | ○ | ○ |
| 9. I am able to make the case when skill mix falls below acceptable levels. | ○ | ○ | ○ | ○ | ○ |
| 10. I am able to access opportunities to actively participate in influencing decisions in my directorate/division. | ○ | ○ | ○ | ○ | ○ |
| 11. I work in a team that encourages everyone's contribution to person-centred care. | ○ | ○ | ○ | ○ | ○ |
| 12. The contribution of colleagues is recognised and acknowledged. | ○ | ○ | ○ | ○ | ○ |
| 13. I am encouraged and supported to lead developments in practice. | ○ | ○ | ○ | ○ | ○ |
| 14. I am supported to do things differently to improve my practice. | ○ | ○ | ○ | ○ | ○ |
| 15. I am able to balance the use of evidence with taking risks. | ○ | ○ | ○ | ○ | ○ |
| 16. I pay attention to the impact of the physical environment on people's dignity. | ○ | ○ | ○ | ○ | ○ |
| 17. In my team we take time to celebrate our achievements. | ○ | ○ | ○ | ○ | ○ |
| 18. I integrate my knowledge of the person into care delivery. | ○ | ○ | ○ | ○ | ○ |

Questionnaire (please tick one response for each question)

| | Never | Rarely | Sometimes | Often | Always |
|---|---|---|---|---|---|
| 19. I work with the person within the context of their family and carers. | ○ | ○ | ○ | ○ | ○ |
| 20. I work with the person to set health goals for their future. | ○ | ○ | ○ | ○ | ○ |
| 21. I try to understand the person's perspective. | ○ | ○ | ○ | ○ | ○ |
| 22. I ensure my full attention is focused on the person when I am with them. | ○ | ○ | ○ | ○ | ○ |
| 23. I strive to gain a sense of the whole person. | ○ | ○ | ○ | ○ | ○ |

© Copyright: Tyagi, Slater, McCance and McCormack 2025.This questionnaire is openly licensed through CC-BY-NC-SA 4.0 license.

For more information, follow this link - https://creativecommons.org/licenses/by-nc-sa/4.0

# THE PERSON-CENTRED PRACTICE INVENTORY – CARE

Thank you for agreeing to complete this questionnaire. Your participation is greatly appreciated.

There are no “right” or “wrong” answers. Please answer each question as accurately as possible. Please take your time and answer ALL of the questions.

The following questions are about you. Please tick one answer for each question. We will not be able to identify you by your answers.

1. Are you? Female ☐
   Male ☐
   Other ☐

2. Your age is 18-24 ☐
   25-44 ☐
   45-54 ☐
   55-65 ☐
   65+ ☐

Please indicate how much you agree or disagree with each of the following statements:

| | | Strongly Disagree | Disagree | Neutral | Agree | Strongly Agree |
|---|---|---|---|---|---|---|
| 1. | Staff try to understand what is important to me | ☐ | ☐ | ☐ | ☐ | ☐ |
| 2. | Staff use my personal experiences to build a relationship with me | ☐ | ☐ | ☐ | ☐ | ☐ |
| 3. | Staff involve me in making decisions about my care | ☐ | ☐ | ☐ | ☐ | ☐ |
| 4. | Staff consider my home environment in meeting my care needs | ☐ | ☐ | ☐ | ☐ | ☐ |
| 5. | Staff give me their full attention when they are with me | ☐ | ☐ | ☐ | ☐ | ☐ |
| 6. | I feel able to say to staff what is important to me | ☐ | ☐ | ☐ | ☐ | ☐ |
| 7. | I feel able to give staff feedback about my experience of being cared for | ☐ | ☐ | ☐ | ☐ | ☐ |
| 8. | Staff ask me about my life | ☐ | ☐ | ☐ | ☐ | ☐ |
| 9. | Staff connect with me as a person | ☐ | ☐ | ☐ | ☐ | ☐ |
| 10. | Staff ask me if I have all the information I need | ☐ | ☐ | ☐ | ☐ | ☐ |
| 11. | When we disagree about my care, staff try to find common ground | ☐ | ☐ | ☐ | ☐ | ☐ |
| 12. | In caring for me, staff use what they know about me as a person | ☐ | ☐ | ☐ | ☐ | ☐ |
| 13. | I feel cared for | ☐ | ☐ | ☐ | ☐ | ☐ |
| 14. | Staff respond compassionately when I am upset or unhappy | ☐ | ☐ | ☐ | ☐ | ☐ |
| 15. | Staff help me to express my concerns about my treatment and care. | ☐ | ☐ | ☐ | ☐ | ☐ |
| 16. | Staff listen to me and hear what I have to say about my care | ☐ | ☐ | ☐ | ☐ | ☐ |
| 17. | Staff understand my family circumstances when caring for me. | ☐ | ☐ | ☐ | ☐ | ☐ |
| 18. | Staff help me to set realistic goals | ☐ | ☐ | ☐ | ☐ | ☐ |

**Telehealth Usability Questionnaire (TUQ) – GP Version**

Instructions:
Please rate your level of agreement from 1-5 with the following statements based on your experience using telehealth.

| Statement | Strongly disagree (1) | Disagree (2) | Neutral (3) | Agree (4) | Strongly agree (5) |
|---|---|---|---|---|---|
| **Usefulness** | | | | | |
| Telehealth improves my ability to deliver quality patient care | | | | | |
| Telehealth allows me to consult with patients as effectively as in-person visits for certain conditions | | | | | |
| Telehealth increases efficiency in my clinical workflow | | | | | |
| Using telehealth improves access to care for my patients | | | | | |
| **Ease of Use and Learnability** | | | | | |
| It is easy for me to learn how to use the telehealth system | | | | | |
| The system is simple and straightforward to operate | | | | | |
| I can use the telehealth platform without needing frequent technical support | | | | | |
| **Interface Quality** | | | | | |
| The interface is clear and easy to navigate during consultations | | | | | |
| The system provides the features I need to manage | | | | | |

| | | | | | |
|---|---|---|---|---|---|
| my consultations effectively | | | | | |
| I can easily access clinical information (e.g. notes, pathology, prescriptions) during telehealth sessions | | | | | |
| **Interaction Quality** | | | | | |
| The audio and video quality are sufficient to conduct a clinical consultation | | | | | |
| I can communicate with patients effectively via telehealth | | | | | |
| I can pick up on visual or non-verbal cues through telehealth when needed | | | | | |
| **Reliability** | | | | | |
| The system is stable and does not crash during use | | | | | |
| I can rely on the telehealth platform to function consistently | | | | | |
| Technical issues (e.g. lags, dropped calls) are minimal and manageable | | | | | |
| **Satisfaction and Future Use** | | | | | |
| I am satisfied with my experience using the telehealth system | | | | | |
| I would prefer to use telehealth regularly for suitable consultations | | | | | |
| I would recommend this telehealth system to other GPs | | | | | |
| I would like further training or support to optimise my telehealth use (Optional exploratory item). | | | | | |

**Telehealth Usability Questionnaire (TUQ) – Patient Version**

Instructions:
Please rate your level of agreement from 1-7 with the following statements based on your experience using the telehealth system.

| No. | Statement | Strongly disagree (1) | Disagree (2) | Somewhat disagree (3) | Neither agree nor disagree (4) | Somewhat agree (5) | Agree (6) | Strongly agree (7) |
|---|---|---|---|---|---|---|---|---|
| 1. | Telehealth improves my access to healthcare services. | | | | | | | |
| 2. | Telehealth saves me time traveling to a hospital or specialist clinic. | | | | | | | |
| 3. | Telehealth provides for my healthcare needs. | | | | | | | |
| 4. | It was simple to use this system. | | | | | | | |
| 5. | It was easy to learn to use the system. | | | | | | | |
| 6. | I believe I could become productive quickly using this system. | | | | | | | |
| 7. | The way I interact with this system is pleasant. | | | | | | | |
| 8. | I like using the system. | | | | | | | |
| 9. | The system is simple and easy to understand. | | | | | | | |
| 10. | This system is able to do everything I would want it to be able to do. | | | | | | | |
| 11. | I can easily talk to the clinician using the telehealth system. | | | | | | | |
| 12. | I can hear the clinician clearly using the telehealth system. | | | | | | | |
| 13. | I felt I was able to express myself effectively. | | | | | | | |
| 14. | Using the telehealth system, I can see the clinician as well as if we met in person. | | | | | | | |
| 15. | I think the visits provided over the telehealth system are the same as in-person visits. | | | | | | | |
| 16. | Whenever I made a mistake using the system, I could recover easily and quickly. | | | | | | | |
| 17. | The system gave error messages that clearly told me how to fix problems. | | | | | | | |

| | | | | | | | | |
|---|---|---|---|---|---|---|---|---|
| 18. | I feel comfortable communicating with the clinician using the telehealth system. | | | | | | | |
| 19. | Telehealth is an acceptable way to receive healthcare services. | | | | | | | |
| 20. | I would use telehealth services again. | | | | | | | |
| 21. | Overall, I am satisfied with this telehealth system. | | | | | | | |

## Patient Satisfaction Questionnaire

Please indicate your level of agreement with the following statements by selecting the appropriate option.

| No. | Statement | Strongly disagree | Disagree | Not sure | Agree | Strongly agree |
|---|---|---|---|---|---|---|
| 1. | How satisfied are you with the effect of your treatment/care? | | | | | |
| 2. | How satisfied are you with the explanations the doctor/health provider has given you about the results of your treatment/care? | | | | | |
| 3. | The doctor/other health professional was very careful to check everything when examining you. | | | | | |
| 4. | How satisfied were you with the choices you had in decisions affecting your health care? | | | | | |
| 5. | How much of the time did you feel respected by the doctor/other health professional? | | | | | |
| 6. | The time you had with the doctor/other health professional was too short. | | | | | |
| 7. | Are you satisfied with the care you received in the hospital/clinic? | | | | | |
