# Supplementary material for: The GRACE video-telehealth project protocol: a mixed-methods study to improve quality, safety and acceptability of video-telehealth in Australian general practice and residential aged care
Source: BMJ Open. 2026 Apr 29;16(4):e110642. doi: 10.1136/bmjopen-2025-110642 (PMC13141005; doi:10.1136/bmjopen-2025-110642)
Supplement: online supplemental file 2 [file bmjopen-16-4-s002.pdf]

INTERVIEW QUESTIONS RACH STAFF

Context

Date of interview: ____

Time of interview: Beginning: _______________ End: ____________________

Total duration of interview: _________________________

Interviewers (initials): _________________

Participant's code: _________________________

Instructions

Hello, my name is (ENTER NAME), I am part of the research team from the University of Sydney. Thank you for agreeing to be interviewed for this Telehealth research project. We are interested in understanding your perceptions of and experiences using Telehealth. This interview should take around 45 minutes.

Is it okay if record the interview?

Do you have any questions before we begin?

To start with, can I ask you to fill in this form? (Give pre interview form if it has not already been completed)

Interview Questions

General impressions of telehealth (TH) and context of use

1. How often would you say you use (or have used) TH with residents in aged care homes? (prompt: multiple times a week, once a month? not very often at all?)
2. Do you record a TH consultation in the patient's file? (Prompt: e.g., emr?)
3. How often would you say you use (or have used) **phone calls** with residents in aged care homes? (prompt: multiple times a week, once a month? not very often at all?)
4. How often would you say you use (or have used) **video** with residents in aged care homes? (prompt: multiple times a week, once a month? not very often at all?)
5. In thinking back to when you last used TH with a resident, did it work well? Yes/No. If yes, what worked well? (e.g., quality care)
6. Can you tell me about any challenges you experienced or have experienced with residents and their TH consultation? (prompt: organisational, people or technological perspectives)
7. Were you able to troubleshoot and overcome any of the challenges? (Prompt: If yes, how? E.g., IT support)

8. Are any of these challenges ongoing? Which ones and why?
9. How do you think this may be remedied or made easier?

Work processes

10. Thinking about the last time you had a TH consultation; can you walk me through the process? What does it usually entail? (prompt: how do you know that your next resident will be a TH style consultation?
11. Is there a different approach based on the technology being used? (follow up: how are they different?)
12. What features or functions might improve the current TH technology you use? (Prompt: processes, policies, education, technology, organisational).
13. Do you think there are any benefits in using TH for RACH residents? If so, what do you think they are? (follow up: if not why?)
14. In what instances do you think that TH can't or shouldn't be used for residents?
15. Have you had (or would you like) any training and/or support when using TH? (follow up: If yes, please describe; if not why do you think?)

Workarounds/adaptations

16. Can you think of a time when you were using TH that you needed to work around/circumvent/adapt to barriers and challenges you encountered?

Prompt examples (only read if needed):

- change the way it was performed, adapt to resident's needs, adjust what you were doing to suit the urgency of the situation, self-organisation when guidelines were inappropriate to solve a situational challenge (*reframing*)
- Cope with unexpected challenges or external demands (*coping*)
- Where you needed to improvise a solution (i*nnovating*)
- Align your practice to a specific resident's need – like a trade-off (*aligning*)

17. Why did you need to use a workaround? (prompt: save time, patient-centred, maintain safety, contextual limitations)
18. Do you need to use workarounds often?
19. If yes, how do they make you feel? (Prompt: competent vs. fatigued)
20. Any other comments?

Recruitment

21. Do you know any residents/carers who may be interested in participating in this research?

Thank you for your time today, it's really appreciated (Give gift card.)
