# Supplementary material for: The GRACE video-telehealth project protocol: a mixed-methods study to improve quality, safety and acceptability of video-telehealth in Australian general practice and residential aged care
Source: BMJ Open. 2026 Apr 29;16(4):e110642. doi: 10.1136/bmjopen-2025-110642 (PMC13141005; doi:10.1136/bmjopen-2025-110642)
Supplement: online supplemental file 3 [file bmjopen-16-4-s003.pdf]

Workshop guide

<table>
<tr><td colspan="2">Workshop topic guide
General Practitioners (GP), GP mangers/staff, RACH staff, residents/carers, consumers</td></tr>
<tr><td>Workshop 1
All stakeholders
Apr 2026
(60 minutes)
Objective: To understand the overall barriers and information requirements to address these</td><td>Welcome and presentation of objectives
Presentation and discussion about Phase 1 findings including member checking to establish agreement (e.g., barriers)
Summarise overall barriers and those related to information requirements (e.g., knowledge, access to information)
Brainstorm some solutions to identified barriers
Participants will be presented with broad questions such as 'which of these barriers could be addressed with an online resource?' and 'what big questions do you have that you would like answered in a digital resource? and asked to write down and rank their top 3 in order of preference (20 minutes)
Discussion and summary of key questions</td></tr>
<tr><td>Workshop 2
2a) GPs, practice mangers, and other staff
2b) RACH staff, including registered nurses, care workers and managers
2c) RACH residents and carers
May-Jun 2026
(60-90 minutes)
Objective: Generate information requirements and how this would look in a digital hub of resources.</td><td>Welcome and presentation of objectives
Participants will be presented with broad questions such as 'In a perfect world, what might be an ideal online education resource in video-telehealth to address these barriers?'
Explore types of online resources needed and what is suitable for them to prioritise and shape the content based on preferences and specific learning needs, ensuring the resulting resources align closely with their educational requirements and enhance overall engagement.
Brainstorming design ideas for online portal: participants to identify educational design features and content, comprehension, presentation, utility, they believed the design solution should have.
Prioritising topics for online portal: Conduct a quick prioritisation exercise, such as dot voting, where participants can vote on the (e.g., TOP THREE) areas they believe should be prioritised in the educational resource.
As a guide, we may explore:
1. Types of resources for education
2. Skills based modules/workshops and topics
3. Point of care resources/pathways
Discuss the structure.</td></tr>
<tr><td>Workshop 3
Consumers
Jun 2026
(60 minutes)
Objective: Ensure that the previous workshop data meets consumers' wants and needs</td><td>Welcome and presentation of objectives
Presentation and discussion about workshop findings including a reflection and "sense" checking to ensure that consumer needs are met</td></tr>
</table>

Workshop guide

**Intervention developed with Medcast (~ 3 months)**	
**Workshop 4**<br>**All stakeholders**<br>**Oct 2026**<br>**(60 minutes)**	**Presenting prototype 1**<br>Participants will be asked questions such as:<br>• What are your thoughts on the materials?<br>• How could the material be improved? Both content and design<br>• Do you think the materials provide sufficient information?<br>• Do you think the most important information can be easily found/accessed in the materials?<br>• Is there a logical order in how the information is presented?<br>• What do you think of infographics/videos?<br><br>Summarise process and thank participants.<br>Discussion with consumers about the prototype portal and whether it meets their needs.
**If needed:**<br>**Interviews with 1-2 subject matter experts within each group and at each iteration of product (~1-3)**<br>**(30 mins)**<br>**Objective: Product development and refinement**<br>**(Oct-Dec 2026)**	**Presenting prototype 2/3**<br>Participants will be asked questions such as:<br>• What are your thoughts on the new prototype?<br>• Could this material be improved? Both content and design<br><br>Summarise process and thank participants.<br>Discussion with consumers about the prototype portal and whether it meets their needs.
