# Supplementary material for: The GRACE video-telehealth project protocol: a mixed-methods study to improve quality, safety and acceptability of video-telehealth in Australian general practice and residential aged care
Source: BMJ Open. 2026 Apr 29;16(4):e110642. doi: 10.1136/bmjopen-2025-110642 (PMC13141005; doi:10.1136/bmjopen-2025-110642)
Supplement: Supplementary file 4 [file bmjopen-2025-110642.reviewer_comments.pdf]

PEER REVIEW HISTORY

BMJ Open publishes all reviews undertaken for accepted manuscripts. Reviewers are asked to complete a checklist review form and are provided with free text boxes to elaborate on their assessment. These free text comments are reproduced below.

ARTICLE DETAILS

Title (Provisional)

Ensuring the quality, safety, acceptability, and sustainability of virtual care with video-telehealth in general practice and residential aged care settings: The GRACE Video-Telehealth project protocol, Phases 1-2

Authors

Carrigan, Ann; Baysari, Melissa T; Luscombe, Georgina; McCormack, Brendan; Von Huben, Amy; Tyagi, Vaibhav; Taggart, Richard; Nash, Jill; Willcock, Simon; Wilkinson, David; Barnett, Stephen; Bonney, Andrew; Rhee, Joel J.; Pearce, Christopher; Robinson, Fiona; Chen, Timothy F; Vagholkar, Sanjyot; Russell, Heather; Chua, Ai-Vee; Shaban, Ramon; Soumya, Soumya; Sengupta, Agnivo; Makeham, Meredith

---

VERSION 1 - REVIEW

**Reviewer**	**1**
**Name**	**Hajilo, Parisa**
**Affiliation**	**Hamadan University of Medical Sciences**
**Date**	**02-Dec-2025**
**COI**	**None**

I have reviewed the manuscript entitled “Ensuring the quality, safety, acceptability, and sustainability of virtual care with video-telehealth in general practice and residential aged care settings: The GRACE Video-Telehealth project protocol, Phases 1–2”, submitted to BMJ Open. Although the topic is important and highly relevant to contemporary virtual-care research, the protocol has several substantial and fundamental limitations that require careful attention. The key issues are summarised below.

1. Major weaknesses in study design coherence and methodological clarity

The study is introduced as a three-phase project; however, Phase 3 lacks almost all essential methodological details, including evaluation criteria, analytic plan, outcome measures, and indicators of effectiveness.

It is unclear how data from Phases 1 and 2 will meaningfully inform the design or evaluation of Phase 3.

The logical alignment between the research questions, methods, and intended outcomes is insufficiently articulated.

2. Insufficient scientific justification and unclear innovation

The manuscript claims to be "the first comprehensive study in Australia," yet provides no systematic comparison with recent national and international literature (2020–2024).

The proposed model of care does not clearly demonstrate how it differs from existing telehealth frameworks, educational programs, or digital care models already implemented in aged care settings.

3. Significant limitations in sample size and sampling strategy

A total of 48 participants is inadequate given the diversity of roles involved (GPs, nurses, managers, RACH staff, residents, carers).

The heterogeneity of participant roles, without stratified planning or subgroup analysis, compromises the credibility and transferability of findings.

Reliance solely on "thematic saturation" is not a sufficient justification for a multi-phase mixed-methods protocol.

4. Ambiguity regarding data collection and data quality

The procedures for extracting data from Services Australia and clinical software systems lack detail; in practice, such datasets often contain delays, inconsistencies, or access restrictions.

No strategy is provided for handling missing data, data inconsistencies, or data quality limitations.

5. Excessive respondent burden for older and vulnerable participants

The simultaneous use of multiple lengthy questionnaires (TUQ, SAPS, PCPI-S, PCPI-C) may impose a high cognitive load and fatigue, especially for older adults and individuals with functional limitations.

No feasibility or pilot testing is mentioned to assess whether these tools are appropriate for this population.

6. Ethical and operational issues insufficiently addressed

The protocol does not clearly explain the process for assessing capacity to consent, particularly for participants with cognitive impairment.

There is no plan to accommodate participants with linguistic, cultural, or cognitive needs.

The collaboration with Medcast may introduce potential conflicts of interest, but this is not adequately discussed.

7. Weaknesses in the data analysis plan

Quantitative analysis is limited to basic descriptive statistics, with no defined statistical models, trend analyses, or evaluation framework for intervention effects.

Key components of rigorous qualitative analysis are missing, including

procedures to reduce researcher bias,

inter-coder reliability, and

reflexivity considerations.

8. Insufficient presentation of tables, figures, and study details

Table 1 and the figures lack complete captions, data sources, and explanatory context.

No timeline, study flow chart, or logic model is provided, despite these being standard expectations under SPIRIT guidelines for protocol reporting.

**Reviewer**	**2**
**Name**	**Stevens, Julie**
**Affiliation**	**Vrije Universiteit Brussel**
**Date**	**15-Dec-2025**
**COI**	**None**

This GRACE video-telehealth study protocol describes a planned two-phase study which scopes resources and models for video-telehealth integration and co-designs a digital resource base for testing in a later phase. The study described is relevant to addressing challenges in access to GP consultations and has potential to promote health equity, provided patients have access to telehealth modalities. The study is overall well described and, the protocol has obtained ethical approval and the study is underway. Hence, my comments mainly relate to points where the manuscript can be clarified in terms of its aims and methods, rather than the conduct of the study itself.

Some recommendations that can hopefully further help clarify the manuscript for the protocol:

1. Introduction: Both paragraph 1 and 2 mention reduced access to GP services, based on the same citation. Perhaps these arguments can be integrated more, or the first paragraph can focus on increasing demand while the second paragraph is dedicated to challenges in access?

2. Introduction: there is mention of resources to support learning new skills to address barriers to the virtual health model of care in persons with e.g. low digital literacy. What are some of these resources? If relevant to the model of care being developed in this study, it would be good to mention them.

3. Introduction: On page 4, there is mention that there is not yet comprehensive Australian research on the impact of safety, quality, … What is meant by quality? Quality of care?

4. Which barriers to implementation, tech and workflow remain for GPs in video telehealth? As in comment 2, it would be useful to mention a few specifically, especially if they are relevant to the model developed in this study.

5. It is mentioned that the study will identify successful models: please specify already from where?

6. Saying implementation of optimized models in RACHS will ultimately lead to safer and higher-quality care for residents is possibly too strong and definitive a statement. Whether this is the outcome is something that should be aimed for and evaluated.

7. The study aims are the first part of the text to introduce person-centred culture, which took me by surprise. It would be helpful if the authors can already address the person-centred component more explicitly in the introduction, as currently the main issue seems to be access (to optimized models).

8. Aim 2 is also potentially confusing in wording: is video telehealth the model of care, or is it a modality (video-telehealth enhanced) to support a broader improved care model that is being developed?

9. The theoretical frameworks are briefly presented and shown in a figure, but there is a lack of detail how its domains precisely inform the work.

10. In the participants and recruitment section on page 9, there is mention of recruiting up to 48 participants across the two phases. I find this wording unclear; does this mean across all potential respondents (GPs, carers, teams, patients/residents, …)? Or per setting, per PHN?

11. How will purposive sampling be implemented, based on which characteristics?

12. Will there be a means to identify which participants (I think mainly patients, then) have had personal experience with video-telehealth and services, since the consultation data will be de-identified for the Phase 1 quantitative data collection? Is there a possibility that participants do not yet have experience with these?

13. For the workshops: will audiences be mixed (e.g. clinicians, residents and carers, consumers)?

14. In data analysis, there is reference to framework analysis (reference 39) under the quantitative data part. Is this correct? Can the authors provide more explanation about how the qualitative data will be quantitatively analysed?

15. Discussion: “Additionally, GPs are becoming more reluctant…” This information was not mentioned in the introduction, I believe; it would be important to mention it there rather than raising another barrier in the discussion?

16. I can’t seem to find the current recruitment or enrollment status. The study is being conducted from October 2025, so as of my reading it has been started recently into its multi-year duration. Have any participants been included so far, or how many GPs/NPs/practices have consented to the Phase 1 data collection?

17. Is this study registered anywhere, or has an evaluation been done as to whether registration is necessary?

Minor grammatical comments:

-Page 3 line 3: comma between “aging” & “where”

-Page 4, line 23”and line 26; page 5 line 30, page 14 line 26: new sentence at “However,”

-Figure 3 caption: should “participatory” be “participating”?

-page 12 line 4-6: please check grammar of this sentence to improve reading flow

-Page 16 line 1: “no know” -> “no known”

VERSION 1 - AUTHOR RESPONSE

We have addressed each of the reviewer comments in the Table below and added two authors who supported the revisions.

Comment	Response
**Reviewer 1**	Thank you for your thorough review to help improve our paper.
Comment from Editor: We note that some of the comments provided by Reviewer 1 are not fully aligned with BMJ Open’s expectations for mixed-method study protocols. Please feel free to disregard the comments relating to the SPIRIT guidelines, small sample size, and lack of Phase 3 methodological details.	Thank you, we have noted this.
1. Major weaknesses in study design coherence and methodological clarity<br><br>The study is introduced as a three-phase project; however, Phase 3 lacks almost all essential methodological details, including evaluation criteria, analytic plan, outcome measures, and indicators of effectiveness.<br><br>The logical alignment between the research questions, methods, and intended outcomes is insufficiently articulated.	Thank you for your valuable feedback.<br><br>A more detailed protocol which includes an economic evaluation (pre/post) will be submitted at a later stage. We have written a sentence in the data analysis section:<br>Page 16, Line 26-

	We have also expanded the phase 3 data collection section and have ensured that the research questions, methods, and intended outcomes are clear.<br>Page 13, Line 4-
It is unclear how data from Phases 1 and 2 will meaningfully inform the design or evaluation of Phase 3.	Phase 1 is scoping the current use of telehealth which will guide the intervention (designed in phase 2). The intervention will be implemented in phase 3 and evaluated. We have expanded sections to improve clarity.<br><br>Page 12, line 25-<br>In Phase 2, the outcomes of Phase 1 will inform the co-design and development of a proposed model of care to support person-centred video-telehealth, and an intervention to facilitate and support video-telehealth utilisation will be developed.<br><br>Page 5, line 31-<br>…we will implement the intervention developed in phase 2…
2. Insufficient scientific justification and unclear innovation<br>The manuscript claims to be "the first comprehensive study in Australia," yet provides no systematic comparison with recent national and international literature (2020–2024).<br><br>The proposed model of care does not clearly demonstrate how it differs from existing telehealth frameworks, educational programs, or digital care models already implemented in aged care settings.	We have removed this point from the Strengths and limitations section and expanded the expected outcomes section in the discussion:<br><br>Page 18, Line 9-

	Unlike existing Australian telehealth initiatives in aged care, which have primarily focused on service expansion or feasibility, this study integrates human factors methodologies, analysis, measurement of outcomes related to person-centred practice to systematically examine safety, workflow alignment, and acceptability of GP-led video-telehealth across diverse RACH settings.
3. Significant limitations in sample size and sampling strategy<br><br>A total of 48 participants is inadequate given the diversity of roles involved (GPs, nurses, managers, RACH staff, residents, carers).<br><br>The heterogeneity of participant roles, without stratified planning or subgroup analysis, compromises the credibility and transferability of findings.<br><br>Reliance solely on "thematic saturation" is not a sufficient justification for a multi-phase mixed-methods protocol.	NA as per Editors instruction
4. Ambiguity regarding data collection and data quality<br><br>The procedures for extracting data from Services Australia and clinical software systems lack detail; in practice, such datasets often contain delays, inconsistencies, or access restrictions.<br><br>No strategy is provided for handling missing data, data inconsistencies, or data quality limitations.	Thank you for highlighting this. We have added more detail to the data collection methods and data analysis.<br>We have added a section to address this:<br><br>Page 15, Line 19-<br><br>**Data quality and missing data management**<br>To ensure completeness and reliability across all

	sources, datasets will undergo structured quality checks, including assessment of missingness, internal consistency (e.g., comparing practice-reported counts with Services Australia totals where feasible), and range checks for implausible values. Where discrepancies or missing data are identified, clarification will be sought from the data provider when possible. Irreconcilable inconsistencies will be documented, and analyses will use complete-case data with sensitivity analyses conducted to assess the impact of missingness. All data quality limitations will be transparently reported and considered when interpreting findings.
5. Excessive respondent burden for older and vulnerable participants<br><br>The simultaneous use of multiple lengthy questionnaires (TUQ, SAPS, PCPI-S, PCPI-C) may impose a high cognitive load and fatigue, especially for older adults and individuals with functional limitations.<br><br>No feasibility or pilot testing is mentioned to assess whether these tools are appropriate for this population.	Our study was co-designed with consumer representatives who ensured that the tasks were not burdensome. We also use scales that have been used and validated in older populations. We have expanded the data collection section to make this clear:<br><br>Page 12, Line 9-<br>To minimise participant burden, questionnaires

	will be administered in flexible formats (secure online platform or paper), and support to complete questionnaires will be offered to residents as needed. Questionnaires selected for this study have been previously validated in older adult populations (refs), and data collection will be paced to align with participant preferences.
6. Ethical and operational issues insufficiently addressed<br><br>The protocol does not clearly explain the process for assessing capacity to consent, particularly for participants with cognitive impairment.<br><br>There is no plan to accommodate participants with linguistic, cultural, or cognitive needs.	Thank you for raising this important point. We agree and have added a section in the participant section:<br><br>Page 10, Line 8-<br>Residents with cognitive impairment (including mild cognitive impairment or a documented diagnosis of dementia) may be invited to participate where their involvement is deemed appropriate by their usual RACH staff, based on day-to-day knowledge of the resident's communication abilities and capacity to engage with the study activities. Capacity to consent will be assessed in accordance with ethical guidelines and the resident's ability to understand, retain and communicate a decision regarding participation. Where a resident is assessed as lacking capacity to provide

	informed consent, participation will only occur with the consent of a legally authorised representative (e.g., a carer, family member, or person exercising lawful authority).<br>To support inclusion of residents from culturally and linguistically diverse (CALD) backgrounds, non-English-speaking residents may participate with the assistance of a carer or family member acting as an informal interpreter, where appropriate and acceptable to the resident. Interpreters will support communication during interviews or questionnaires but will not influence responses. The voluntary nature of participation will be emphasised, and residents and carers may withdraw at any time without consequence.
7. The collaboration with Medcast may introduce potential conflicts of interest, but this is not adequately discussed.	Thank you for highlighting this. To avoid a conflict of interest, Medcast will not be involved in the data analysis and interpretation phase of the project. A sentence has been added to the Phase 2 data collection section:<br><br>Page 14, line 8-<br>Medcast will only be involved in the development of the

	intervention, not its evaluation or data analysis.
8. Weaknesses in the data analysis plan<br><br>Quantitative analysis is limited to basic descriptive statistics, with no defined statistical models, trend analyses, or evaluation framework for intervention effects.<br><br>Key components of rigorous qualitative analysis are missing, including procedures to reduce researcher bias, inter-coder reliability, and reflexivity considerations.	These sections have been expanded with more detail.<br><br>Page 15, Line 15-<br>**Quantitative analysis**<br>For this project, we will map the person-centred practice survey data to the person-centred framework (see Fig 1).<br><br>Page 16, Line 8-<br>**Qualitative analysis**<br>In Phases 1 and 3, to enhance rigour, two researchers will independently code an initial subset (~20%) of transcripts using an iteratively developed codebook. After open coding, the team will meet to compare generated codes, resolve discrepancies by consensus, and refine codes. The remaining transcripts will be double-coded where codes are emergent or complex; otherwise, a single-coder approach will be used with scheduled peer debriefs and spot-checks to ensure consistent application. Reflexive memos will accompany each analytic cycle to make researcher assumptions explicit and

	to reduce bias in development of themes.<br><br>Page 16, Line 26-<br>A detailed plan for the analysis will be submitted as a separate protocol which includes economic outcomes where we will assess the costs and outcomes associated with the video-telehealth intervention.
9. Insufficient presentation of tables, figures, and study details<br><br>Table 1 and the figures lack complete captions, data sources, and explanatory context.<br><br>No timeline, study flow chart, or logic model is provided, despite these being standard expectations under SPIRIT guidelines for protocol reporting.	NA as per Editors instruction
**Reviewer 2**	Thank you for your thorough review to help improve our paper.
**1.** Introduction: Both paragraph 1 and 2 mention reduced access to GP services, based on the same citation. Perhaps these arguments can be integrated more, or the first paragraph can focus on increasing demand while the second paragraph is dedicated to challenges in access?	Thank you for this suggestion. The second paragraph has been merged with the first with a focus on reduced access to GPs. We have also added as sentence to improve clarity:<br><br>Page 3, Line 9-
2. Introduction: there is mention of resources to support learning new skills to address barriers to the virtual health model of care in persons with e.g. low digital literacy. What are some of these resources? If relevant to the model of care being developed in this study, it would be good to mention them.	We have added to this sentence as this is relevant for our study:<br>Page 4, Line 8-<br>To overcome these barriers, educational resources such as training on care delivery modes to support the learning of

	new skills were recommended.
3. Introduction: On page 4, there is mention that there is not yet comprehensive Australian research on the impact of safety, quality, … What is meant by quality? Quality of care?	Thank you for noting this. The sentence has been changed to make clearer we are referring to quality of care:<br>Page 4, Line 21-<br>However, there is no comprehensive Australian research that directly assesses the impact of GP-led video-telehealth on safety, quality **of** care…
4. Which barriers to implementation, tech and workflow remain for GPs in video telehealth? As in comment 2, it would be useful to mention a few specifically, especially if they are relevant to the model developed in this study.	We have expanded this sentence with relevant examples:<br><br>Page 4, Line 24-<br>…however, barriers related to implementation (e.g., insufficient training), technology (e.g., inadequate internet connectivity) and workflow (e.g., unpredictable delays when calls are not coordinated well) remain.
5. It is mentioned that the study will identify successful models: please specify already from where?	We have changed this sentence as it is not yet known what a successful model is:<br>Page 5, Line 7-<br>This study will identify current telehealth models (with or without video), optimise them, and provide the necessary resources and guidance for their effective implementation in RACHS, possibly supporting…

6. Saying implementation of optimized models in RACHS will ultimately lead to safer and higher-quality care for residents is possibly too strong and definitive a statement. Whether this is the outcome is something that should be aimed for and evaluated.	Our outcome measures include quality and safety indicators from RACHs (e.g., hospital transfers). We have made this clearer in the data collection section and revised our expected outcomes in the introduction and discussion.<br><br>Page 5, Line 10-<br>…possibility supporting safer and higher-quality care for residents.<br><br>Page 11, Line 24-<br>Clinical quality and safety indicators (e.g., hospital transfers, falls) from RACHs will also be recorded. No identifiable resident data will be shared.<br><br>Page 18, line 13-<br>…our co-designed model **may** help reduce inequities and primary care shortages
7. The study aims are the first part of the text to introduce person-centred culture, which took me by surprise. It would be helpful if the authors can already address the person-centred component more explicitly in the introduction, as currently the main issue seems to be access (to optimized models).	We have changed this to person-centred practice (PCP) which aligns with the theoretical framework that we are using. A paragraph introducing this has been added to the introduction.<br><br>Page 4, Line 28-
8. Aim 2 is also potentially confusing in wording: is video telehealth the model of care, or is it a modality (video-telehealth enhanced) to support a broader improved care model that is being developed?	We agree this is confusing as video-telehealth is the modality supporting the model. This has been changed:

	Page 5, Line 25-<br>To co-design and develop a best practice model of care enhanced by video-telehealth that includes a digital hub of resources and support materials, using codesign methods and implementation strategies that drive adoption of person-centred video-telehealth in GP/RACH settings.
9. The theoretical frameworks are briefly presented and shown in a figure, but there is a lack of detail how its domains precisely inform the work.	A paragraph has been added to the introduction.<br><br>Page 4, Line 28-<br>Healthcare systems are complex and multifaceted. Understanding the barriers related to implementation, technology and workflow in healthcare, therefore, requires a whole system and human-centred approach. Human Factors (HF), the study of how people interact within systems, provides a way to understand the barriers to video-telehealth by considering the work system elements involved (technology, tasks, people, environment), processes and outcomes[27]. For example, barriers related to usability of technology[28]. Additionally, person-centred practice (PCP) ensures that care is respectful, individualised, and responds to a

	person's needs [29]. Together, adopting a HF and PCP approach creates digital health environments that are safe, efficient, and meaningful which is crucial in aged care settings.<br>We have also added the RE-AIM framework which will guide evaluation in Phase 3, to the theoretical framework section:<br><br>Page 8, Line 10-<br>In Phase 3, we will be guided by the RE-AIM framework an established model that has been widely used in health service research to assess real-world impact of the intervention (ref). The framework proposes assessing Reach (e.g., number of residents involved in the intervention), Effectiveness (e.g., resident experience), Adoption (e.g., number of GPs using video-telehealth), Implementation (e.g., training required), and Maintenance (e.g., practices that embed video-telehealth into routine workflows).
10. In the participants and recruitment section on page 9, there is mention of recruiting up to 48 participants across the two phases. I find this wording unclear; does this mean across all	Thank you for noticing this. We have updated this sentence and increased the number:

potential respondents (GPs, carers, teams, patients/residents, …)? Or per setting, per PHN?	Page 9, Line 23-<br>We will recruit up to 72 participants across all potential respondents (GPs, NPs, managers, RACH staff, residents) and PHNs accounting for study attrition.
11. How will purposive sampling be implemented, based on which characteristics?	We have added to this section:<br><br>Page 9, Line 16-<br>We will aim to ensure representation across key stakeholder groups involved in video-telehealth delivery and receipt of care. Across all three phases, participants will include GPs and NPs, practice managers, RACH staff, including registered nurses, managers, and carers or family members. While exact numbers per group will remain flexible to allow sampling based on availability and relevance, we anticipate approximately half of participants will be healthcare providers (GPs/NPs and practice or RACH staff) and half residents and carers. In line with previous research, purposive sampling will be used to achieve diversity in professional roles, care settings, and geographic regions, rather than

	statistical representativeness.
12. Will there be a means to identify which participants (I think mainly patients, then) have had personal experience with video-telehealth and services, since the consultation data will be de-identified for the Phase 1 quantitative data collection? Is there a possibility that participants do not yet have experience with these?	A study inclusion is that resident participants have had experience using telehealth (not necessarily video). They will be identified when the study is advertised by a staff member. We have updated this section:<br><br>Page 10, Line 6-<br>Eligible residents will be required to be able to provide informed consent, be aged 65 years or more and have had experience with a telehealth consultation.
13. For the workshops: will audiences be mixed (e.g. clinicians, residents and carers, consumers)?	The workshops will be combined and individual and a clarifying paragraph has been added with reference to Fig 5.<br><br>Page 14, Line 9-<br>Workshops will commence with an information session for all groups where we will present the findings from Phase 1. Individual groups and a consumer workshop will follow, with all groups coming together again after the first iteration of the intervention has been designed (Fig. 5).
14. In data analysis, there is reference to framework analysis (reference 39) under the quantitative data part. Is this correct? Can the authors provide more explanation about how the qualitative data will be quantitatively analysed?	The framework analysis involves mapping the quantitative survey data to the PCPF. We have added a sentence to improve clarity:

	Page 15, Line 15-<br>For this project, we will map the PCP scores to the PCPF[39] (see Fig. 1).
15. Discussion: "Additionally, GPs are becoming more reluctant…" This information was not mentioned in the introduction, I believe; it would be important to mention it there rather than raising another barrier in the discussion?	This sentence has been changed in the discussion.<br><br>Page 17, Line 17-<br>Additionally, barriers related to significant financial costs in visiting RACH which are not met by Medicare, may mean that GPs are becoming more reluctant to provide 'in person visits'.<br><br>A sentence has also been added to the introduction:<br><br>Page 3, Line 9-<br>Additionally, the substantial out-of-pocket costs associated with visiting RACHs, costs that are not covered by Medicare, may be contributing to growing GP reluctance to provide in-person visits.
16. I can't seem to find the current recruitment or enrolment status. The study is being conducted from October 2025, so as of my reading it has been started recently into its multi-year duration. Have any participants been included so far, or how many GPs/NPs/practices have consented to the Phase 1 data collection?	Yes, data collection has commenced. We have not included these details (results) in the paper as a protocol paper.
17. Is this study registered anywhere, or has an evaluation been done as to whether registration is necessary?	This study was evaluated by the University of Sydney who concluded it was not a clinical trial and therefore does not require registration.
18. Page 3 line 3: comma between "aging" & "where"	This has been amended

19.	Page 4, line 23"and line 26; page 5 line 30, page 14 line 26: new sentence at "However,"	These have been amended
20.	Figure 3 caption: should "participatory" be "participating"?	This has been amended
21.	page 12 line 4-6: please check grammar of this sentence to improve reading flow	This has been amended
	Page 16 line 1: "no know" -> "no known"	This has been amended

We hope our manuscript is now suitable for publication and look forward to hearing an outcome soon.

VERSION 2 - REVIEW

Reviewer 2

Name Stevens, Julie

Affiliation Vrije Universiteit Brussel

Date 16-Apr-2026

COI

I very much appreciate the authors engaging with my feedback. The responses addressed my questions and remarks well and have improved the clarity of the manuscript.

Only a very minor comment:

-"For example, barriers related to usability of technology." (P5 line 1) - The additional paragraph is informative, but this particular sentence is incomplete.
